# Long-term presence of autoantibodies in plasma of cured leprosy patients

**DOI:** 10.1038/s41598-022-27256-x

**Published:** 2023-01-05

**Authors:** Xi Yang, Hua Dong, Yi-Qun Kuang, Xiu-Feng Yu, Heng Long, Chun-Yu Zhang, Dong Wang, Deng-Feng Zhang, Yu-Ye Li

**Affiliations:** 1 Department of Dermatology, First Affiliated Hospital of Kunming Medical University, Kunming, 650032 Yunnan China; 2 NHC Key Laboratory of Drug Addiction Medicine, First Affiliated Hospital of Kunming Medical University, Kunming, 650032 Yunnan China; 3 Wenshan Institute of Dermatology, Wenshan, 663000 Yunnan China; 4 Key Laboratory of Animal Models and Human Disease Mechanisms of the Chinese Academy of Sciences and Yunnan Province, Kunming Institute of Zoology, Chinese Academy of Sciences, Kunming, 650223 Yunnan China

**Keywords:** Immunology, Autoimmunity, Microbiology, Bacteria, Bacterial host response

## Abstract

Autoantibodies have been detected in leprosy patients, indicating that infection with *M. leprae* may lead to autoimmune disorders. However, whether autoimmune response last until patients are cured is unknown. Knowing the autoimmune response in cured leprosy patients is essential to identify whether symptoms are caused by leprosy itself or by other immune-related diseases. This knowledge is essential for the ongoing health management in cured leprosy patients where autoimmune disorders still exist. In our study, we selected six autoantibodies, including anticardiolipin antibody of IgG (ACA), anti-nuclear antibody (ANA), extractable nuclear antigen antibody (ENA), anti-streptolysin O (ASO), anti-double stranded DNA antibody (dsDNA), and rheumatoid factor (RF), that had been reported in leprosy patients as typical autoantibodies. We tested the six typical autoantibodies combined with LACC1, which encodes a protein associated with autoimmune disease such as Crohn’s disease and is also the susceptible gene conferring leprosy risk, in cured leprosy patients through ELISA to assess the cured patient’s immune status. We observed high positive rates of autoantibodies in cured leprosy patients, and the average plasma levels of five (ACA, ANA, ENA, ASO, and RF) out of the six autoantibodies were significantly higher in cured leprosy patients than in controls. The positive detection of autoantibodies is independent of the recovery period. Moreover, the level of these autoantibodies showed a strong positive correlation with the level of LACC1 in both controls and cured patients. This study showed that there is long-term autoimmunological activation in leprosy patients, even after decades of recovery. Autoimmune responses may influence the development and prognosis of leprosy. Special care should be given to posttreatment or cured leprosy patients regarding long-term autoimmunological activation.

## Introduction

Leprosy is an ancient chronic infectious disease caused by Mycobacterium leprae (*M. leprae*) which mainly infects skin and peripheral nerve. The pathogen *M. leprae* has a highly degraded genome and is a stringent host-dependent intracellular parasite^1^. The incubation period of leprosy following *M. leprae* infection ranges from months to decades. And there are various clinical subtypes including tuberculoid (TT), borderline tuberculoid (BT), borderline (BB), borderline lepromatous (BL), and lepromatous leprosy (LL)^2^. Both the incubation period and clinical manifestations are determined by the host immunological response and genetic background. Indeed, immunological abnormalities are prevalent in leprosy, since systemic lupus erythematosus-like and rheumatism-like autoimmune manifestations are often observed in leprosy patients^3,4^, and the positive of autoantibodies like anticardiolipin antibody of IgG (ACA), anti-nuclear antibody (ANA), extractable nuclear antigen antibody (ENA), anti-Streptolysin O (ASO), anti-double stranded DNA antibody (dsDNA) and rheumatoid factor (RF) had been reported in leprosy patients^5–8^. We also have previously shown that genetic variations of typical autoimmune diseases relevant genes^9^, especially LACC1^10^, which encoded protein associated with autoimmune disease such as Crohn’s disease is also the susceptible gene confer leprosy risk^8,11,12^. It is evident that immunological abnormalities, especially autoimmune reactions, are involved during the onset and development of leprosy^13^, indicated that infected with *M. leprae* may lead to immune disorders. But whether the immune disorders lead by *M. leprae* may continuous exist remains unclear, which is important to leprosy prognosis as patients with immune disorders need ongoing health management. In our study, we selected ACA, ANA, ENA, ASO, dsDNA, and RF that had been reported in leprosy patients as typical autoantibodies, and tested the six typical autoantibodies combined with LACC1 in cured leprosy patients through ELISA to assess the cured patient's immune status.

## Results

### Demographic and clinical characteristics

There were 198 cured leprosy patients (63 females) enrolled. The age of those patients ranged from 16 to 98 years, with a median age of 58 years old; the onset age ranged from 5 to 75 years, with a median onset age of 25.0 years old. The average year of plasma collection after completing treatment was 26.35 ± 15.47 years, which ranged from less than 1 year to more than 50 years. There were 3 subjects having a history of relapse, and suffered from leprosy reactions. Of the 198 cured patients, 58 had no disability, 43 had different grades of disability, and 97 had missing information of disability. Among the 198 individuals, 73 patients were treated with dapsone; 32 were treated with dapsone and rifampicin; and 90 were treated with dapsone, rifampicin and clofazimine; the treatment of 3 cases was missed. The distribution of demographic and clinical characteristics among leprosy subtypes was shown in Table 1.

**Table 1:** Demographic and clinical characteristics of individuals enrolled in this study.

	Leprosy (N = 198)						Control (N = 101)
	TT (N = 42)		BT (N = 43)	BB (N = 19)	BL (N = 59)	LL (N = 35)	
Mean age (range, years)	58(47, 65)						57.0(48.0, 66.0)
Mean onset age (range, years)	25.0 (17.0, 33.3)						–
**Gender**							
Male	23		31	15	44	22	69
Female	19		12	4	15	13	32
**Treatment**							
Dapsone	21		15	4	11	22	–
Dapsone + Rifampicin	20		11	0	1	0	–
Dapsone + Rifampicin + Clofazimine	1		17	14	45	13	–
**Disability**							
Without disability	11		13	6	19	9	–
Grade I	0		1	0	2	1	–
Grade II	9		12	2	10	6	–
**Post-treatment (Years)**	26.35 ± 15.47						–
< 1	1		2	1	3	0	–
1–10	4		7	5	11	6	–
11–20	8		8	7	14	3	–
21–30	6		12	2	17	3	–
31–40	4		6	4	8	8	–
41–50	12		7	0	6	13	–
> 50	7		1	0	0	2	–

### High positive rate of plasma autoantibodies in cured leprosy patients

We measured the levels of ACA-IgG, ENA-Ab, ASO-Ab, RF-IgA, ANA-Ab, dsDNA-Ab, and LACC1-Ab in the plasma of patients and controls. Since normal individuals have low levels of autoantibodies, an individual with a plasma level of an autoantibody higher than the mean + 2 SD (standard deviation) level of that of the control group was defined as positive for the autoantibody, as based on the manufacturer’s instructions for the ELISA kit. The concentration of ANA > 87.3 ng/mL, ACA-IgG > 43.6 ng/mL, dsDNA > 16.7 ng/mL, ASO > 329.9 U/L, ENA-Ab > 87.4 ng/mL, LACC1 > 3.7 ng/mL, RF > 3.0 ng/mL were thus defined as positive, respectively. As such, higher positive rates of all six detected autoantibodies were observed in cured leprosy patients: 91 (46.0%), 30 (15.2%), 74 (37.4%), 55 (27.8%), 95 (48.0%), and 26 (13.1%) patients were positive for ACA, ENA, ASO, ANA, RF, and dsDNA, respectively; while the positive rate of these autoantibodies ranges around 3–5% in normal controls. There were no significant differences in LACC1, ACA, ENA, ASO, ANA, dsDNA and RF concentrations between different leprosy subtypes (*P* > 0.05). Intriguingly, in the BB subtype, we observed the highest positive rate of ACA but the lowest positive rates of ENA, ASO and ANA (Table 2). In contrast to previous findings in the course of the disease showing that ACA-IgG was significantly higher in lepromatous leprosy and multibacillary patient subgroups^5^, no extremely higher positive rate was observed for lepromatous leprosy in our study. Notably, the positive rates of ASO (45.8%) and RF (61.0%) were extremely high in the BL subtype.

**Table 2:** Positive rate of ACA-IgG, ENA-Ab, ASO-Ab, RF-IgA, ANA-Ab, dsDNA-Ab, LACC1-Ab in the plasma of cured leprosy patients and controls.

	Control (N = 101)	Leprosy *per se* (N = 198)	χ2	P	TT^a^ (N = 42)	BT (N = 43)	BB (N = 19)	BL (N = 59)	LL (N = 35)	χ2	P
Female	32 (31.6%)	63 (31.8%)			19 (45.2%)	12 (27.9%)	4 (21.1%)	15 (25.4%)	13 (37.1%)		
ACA + ^b^	5 (5.0%)	91 (46.0%)	13.818	0.000^d^	12 (28.6%)	22 (51.2%)	***11 (57.9%)***^c^	28 (47.5%)	18 (51.4%)	7.146	0.128
ENA +	3 (3.0%)	30 (15.2%)	11.589	0.001^d^	7 (16.7%)	7 (16.3%)	*1 (5.3%)*	***10 (16.9%)***	5 (14.3%)	1,737	0.785
ASO +	4 (4.0%)	74 (37.4%)	40.442	0.000^d^	15 (35.7%)	18 (41.9%)	4 (21.1%)	***27 (45.8%)***	10 (28.6%)	5.514	0.238
ANA +	4 (4.0%)	55 (27.8%)	34.579	0.000^d^	11 (26.2%)	***16 (37.2%)***	1 (5.3%)	17 (28.8%)	10 (28.6%)	6.803	0.147
RF +	3 (3.0%)	95 (48.0%)	52.383	0.000^d^	17 (40.5%)	23 (53.5%)	7 (36.8%)	***36 (61.0%)***	12 (34.3%)	9.062	0.060
dsDNA +	5 (5.0%)	26 (13.1%)	5.190	0.023^d^	7 (16.7%)	4 (9.3%)	2 (10.5%)	***10 (16.9%)***	3 (8.6%)	2.518	0.641

^a^TT, tuberculoid leprosy, BT, borderline tuberculoid leprosy; BB, mid-borderline leprosy; BL, borderline lepromatous leprosy; LL, lepromatous leprosy;

^b^ +, positive, an individual with the plasma level of an autoantibody higher than the mean + 2 SD (standard deviation) level of that of the control group was defined as positive for the autoantibody.

^c^Highest rate in the five subtypes were marked in bold and italic.

^d^Comparisons of the positive rate of each autoantibody between control and patients groups were conducted using correct Chi-Squared Test of four-fold table, in five subtypes were conducted using Chi-Squared Test of contingency table. A *P* value < 0.05 was considered to be statistical significance.

### Long-term presence of plasma autoantibodies in cured leprosy patients

**Figure 1:**
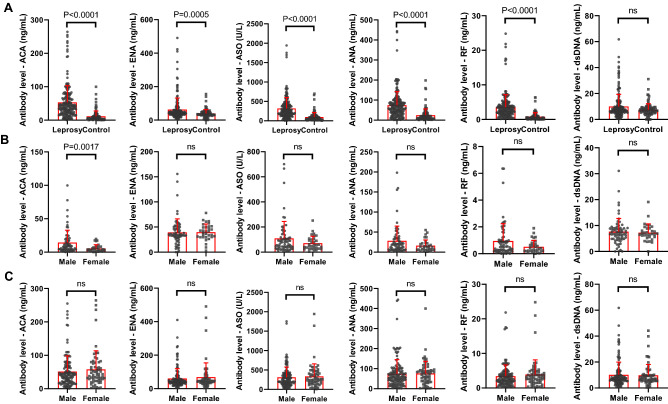
Circulating levels of autoantibodies in cured leprosy patients and controls. (**A**) Comparison of the levels of the autoantibodies in plasma of cured leprosy patients with those of healthy controls. (**B**) Sex-specific level of the autoantibodies in plasma of controls. (**C**) Sex-specific level of the autoantibodies in plasma of cured patients. ACA, anticardiolipin IgG antibody; ENA, anti-extractable nuclear antigen antibody; ASO, human anti-streptolysin O; ANA, antinuclear antibody; RF, human rheumatism factor; dsDNA, anti-double-stranded DNA antibody. Comparisons of the level of each autoantibody in two groups were conducted using the non-parametric Mann–Whitney test. ns: not significant.

**Figure 2:**
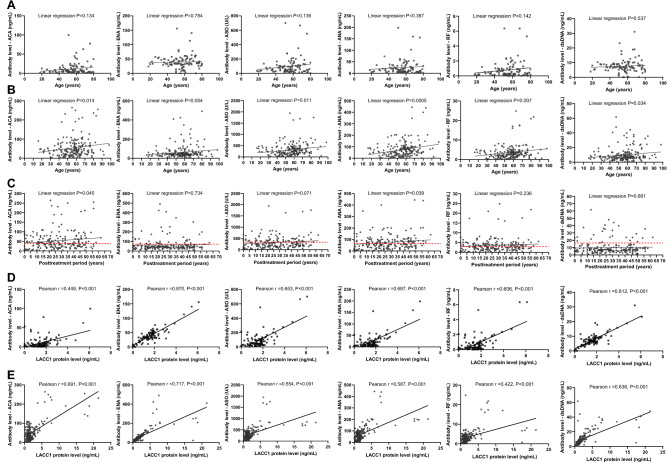
Correlation of the levels of autoantibodies with aging, posttreatment period, and LACC1 protein level. (**A**) Alteration of the autoantibodies in plasma of controls along age (years). (**B**) Alteration of the autoantibodies (ng/ml) in plasma of cured patients along age (years). (**C**) Lifelong presence of the autoantibodies in plasma of cured patients. whereas the alteration of the autoantibodies along time of post-treatment (years) was measured by linear regression. (**D**) Correlation of the levels of the autoantibodies with the level of LACC1 protein (ng/ml) in plasma of controls. (**E**) Correlation of the levels of the autoantibodies with the level of LACC1 protein (ng/ml) in plasma of cured patients. ^*^In linear regression analysis, there is linear correlation when *P* < 0.05. In Pearson correlation analysis, there is correlation when *P* < 0.05, and the correlation is negative when R < 0, positive when R > 0.

In addition to the positive rate, the average levels of these autoantibodies, ACA, ANA, ENA, ASO, and RF, were significantly higher in the plasma of the patient group than in that of the controls (Fig. 1A). Sex had no effect on the plasma levels of these autoantibodies in either controls or patients, except for ACA in controls (Fig. 1B,C). Age had no effect on the plasma levels of these autoantibodies in controls (Fig. 2A), whereas the plasma levels of all six autoantibodies were positively related to age in patients (Fig. 2B). We also found that there was no statistically significant difference in autoantibody concentrations between different disability levels or treatments. Nevertheless, there were weak correlations between the levels of the autoantibodies and the recovery time period (Fig. 2C): the autoantibodies presented in both subjects in the period of onset or subjects in the period after decades of recovery (*P* = 0.045, 0.734, 0.071, 0.039, 0.236, 0.661 for ACA, ENA, ASO, ANA, RF and dsDNA respectively).

### Positive correlation between the levels of autoantibodies and the level of the LACC1 protein

**Figure 3:**
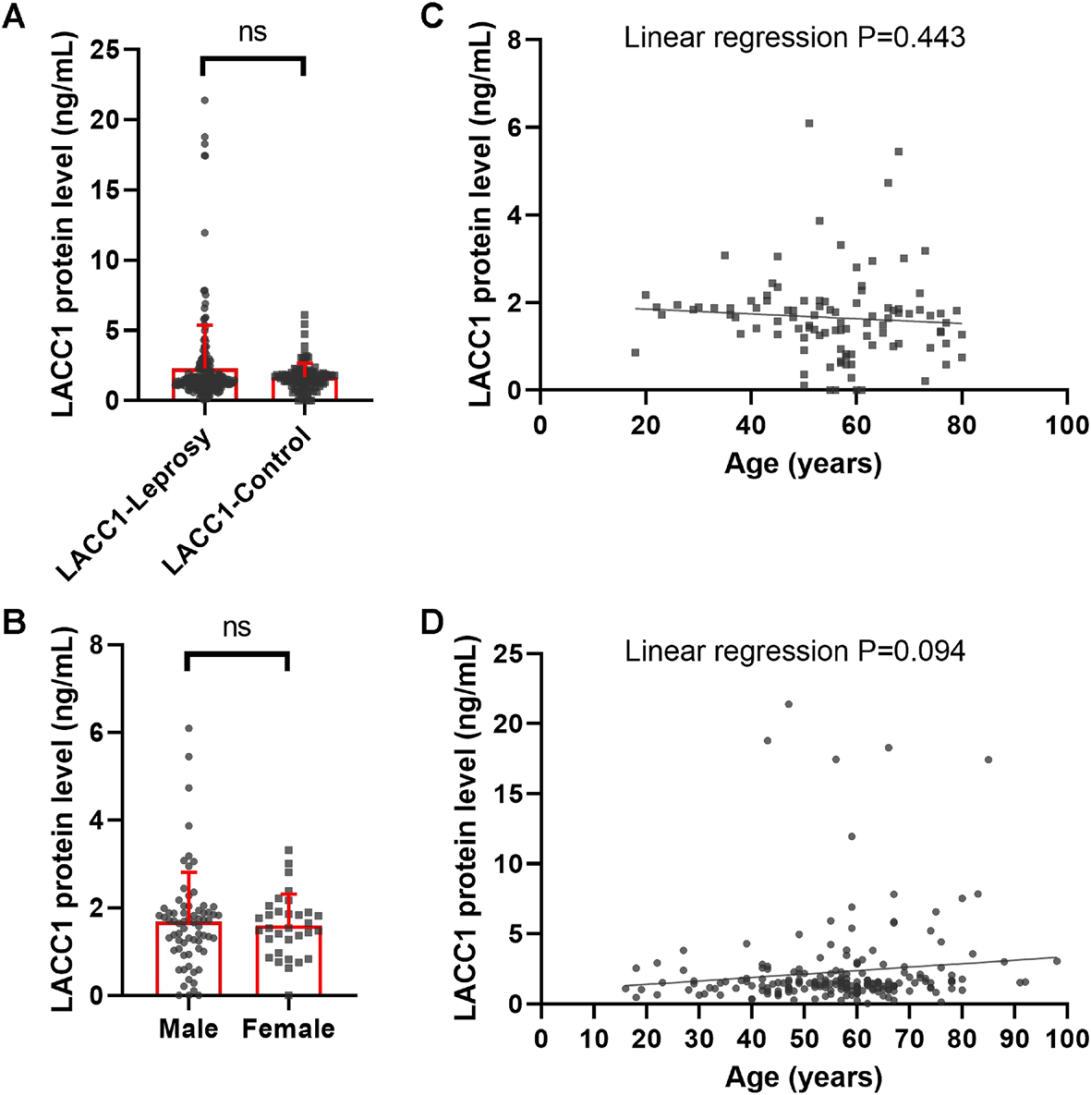
Circulating level of LACC1 protein in cured leprosy patients and controls. (**A**) Comparison of the LACC1 protein level (ng/ml) in plasma of cured leprosy patients with that of healthy controls. (**B**) Sex-specific level of LACC1 (ng/ml) in plasma of controls. (**C**), Alteration of the LACC1 protein (ng/ml) in plasma of controls along age (years). (**D**) Alteration of the LACC1 protein (ng/ml) in plasma of cured patients along age (years).

As LACC1 is a master regulator in autoimmune diseases, we investigated whether the autoantibody levels were related to LACC1 protein levels. We found that the protein level of LACC1 was similar in cured patients and controls, and the level of LACC1 protein increased slightly with age in patients but remained consistent with ageing in controls (*P* = 0.443, 0.094 in control group and patient group, respectively) (Fig. 3). Intriguingly, the levels of all six autoantibodies were strongly related to the protein level of LACC1 in both controls and patients (Fig. 2D,E), supporting the master role of LACC1 in autoimmunity regardless of disease status (Pearson R = 0.448, 0.870, 0.653, 0.687, 0.606, 0.812, *P* < 0.001 of ACA, ENA, ASO, ANA, RF and dsDNA in control group respectively; Pearson R = 0.691, 0.717, 0.554, 0.587, 0.422, 0.636, *P* < 0.001 of ACA, ENA, ASO, ANA, RF and dsDNA in patients group respectively).

## Discussion

The onset and development of leprosy is dependent on host‒pathogen interaction. Since *M. leprae* could be latent for many years before the clinical manifestations, the autoimmune response might be involved in the course of leprosy. Indeed, we and others have shown that genetic variants within autoimmune-related genes and pathways contribute to the development of leprosy^14^. Clinical observations have shown that *M. leprae* infection might mimic the performance of systemic lupus erythematosus^4,15^ and trigger the reactivation of lupus. Numerous studies have reported autoimmune responses during the course of leprosy^13,16^. However, it is unclear whether the autoimmune response occurs before the onset of leprosy or whether autoimmune activation is just an effect of bacterial infection.

We detected the circulating concentrations of six typical autoantibodies in cured leprosy patients with different recovery periods and found that the concentrations of these autoantibodies were significantly higher in cases than in controls. Considering that different methods and materials may affect the results, we defined individuals with a plasma level of an autoantibody higher than the mean + 2 SD (standard deviation) level of that of the control group as positive according to the ELISA kit instructions used. The positive rate of these autoantibodies ranges from 13.1 to 48.0%, which was consistent with previously findings in patients with present illness^6,8,17–19^. Our results showed that age had no effect on the plasma levels of these autoantibodies in controls, whereas in patients the plasma levels of these autoantibodies increase with age, which suggests that the severity of autoimmune disorders caused by *M. leprae* may be age-related. We also found that there was no statistically significant difference in autoantibody concentrations between different disability levels or treatments. This result indicated that the immune status of cured leprosy patients may not be connected to the severity of disease and drugs. However, the levels of dsDNA and ANA concentrations were higher in the BT group than in the BB group. In the BB subtype, we observed the highest positive rate of ACA but the lowest positive rates of ENA, ASO, ANA, and RF. BB leprosy is an intermediate subtype of leprosy, with a lower bacterial load than LL leprosy and a higher bacterial load than TT leprosy, and the immune response is stronger than LL leprosy and weaker than TT leprosy. The highest and lowest immune antibodies in BB leprosy suggest that the cause of the immune disorder may not be solely due to bacterial or host immunity. Notably, we observed the presence of autoantibodies in both subjects in the period of onset and subjects in the period after decades of recovery, indicating a long-term presence of autoantibodies in cured leprosy patients. Since the cured patients haven’t suffered from any autoimmune-related symptoms, the presence of autoantibodies might be a result of the interaction between *M. leprae* and the immune system of the host. As our analysis showed that there is no significant correlation between disability and the presence of autoantibodies, what leads to the raise of autoantibodies, and what’s the consequence of the autoantibodies, remained to be open questions. This phenomenon might be explained by two possibilities: (1) those subjects suffering from leprosy are more susceptible to autoimmune activation or autoimmune complications, and the presence of autoantibodies in these individuals is the result of genetic predisposition rather than the effect of *M. leprae* infection. (2) There are continuous immune activations in cured patients due to unknown reasons or potential latent pathogens. For the first possibility, we observed that the levels of all six autoantibodies were strongly related to the protein level of LACC1, which is a key protein in multiple autoimmune-mediated diseases^20^, in both controls and patients, indicating that the genetic predisposition to autoimmunity could partially explain the high positive rate and level of autoantibodies of cured patients. As there were too less relapse and leprosy reaction happened in enrolled patients, we can’t effectively analyze whether recurrence and leprosy reaction affected these autoantibodies. Therefore, the presence of autoantibodies decades after recovery might be the result of the long-term effect of *M. leprae* infection. Additionally, we cannot rule out the possibility that dead *M. leprae* or its debris might trigger the immune system since it is common to find acid-fast bacilli in skin smears even after completion of multidrug therapy.

## Conclusions

While the presence of autoantibodies in current leprosy patients might partially explain autoimmune-related complications, the presence of autoantibodies in cured patients indicates a long-term effect of *M. leprae* infection on the host immune status. Autoimmunological activation might indicate incomplete treatment of the disease or may act as an inducer for subsequent autoimmune conditions. In both cases, special care should be given regarding long-term autoimmunological activation to both posttreatment and cured leprosy patients.

## Methods

### Demographic information and plasma sample collection

*Demographic Information* We recruited 198 clinically cured leprosy patients (females: 31.8%, mean age (range): 56.2 (16–98) years old) from Yunnan Province, Southwest China. Leprosy diagnosis was established based on the clinical signs and symptoms, skin smears, skin biopsy, and neuro-physiologic examinations when necessary. Patients were considered as cured after they had completed treatment. Epidemiological and clinical information regarding the age at onset, date of discovery, family history of infection, clinical diagnosis, subtype of leprosy, bacterial index, treatment, relapse, reaction, and cure date were recorded, and patients who had coexisting diseases, especially autoimmune diseases, were excluded. A total of 101 healthy subjects from the Physical Examination Center of the First Affiliated Hospital of Kunming Medical University (females: 31.6%, mean age (range): 55.5 (20–80) years old) were enrolled as the control group.

*Plasma samples* Plasma samples of each individual were collected from venous blood with EDTA tubes and underwent a standard venipuncture procedure. The blood was centrifuged for 20 min at 3000 R/min, 3 h after collection, and the upper plasma layer was stored in a 1.5 ml EP tube. All samples were stored at − 80 °C until analysis.

### Plasma ELISA

*Materials* Six typical autoantibodies, including the anticardiolipin IgG antibody (ACA-IgG, Jianglai Bio, JL10574), antinuclear antibody (ANA, Jianglai Bio, JL10653), anti-extractable nuclear antigen antibody (ENA, Jianglai Bio, JL11931), anti-double-stranded DNA antibody (dsDNA, Jianglai Bio, JL37834), human anti-streptolysin O (ASO, Jianglai Bio, JL10053), and human rheumatism factor (RF, Jianglai Bio, JL28325), were measured by enzyme-linked immunosorbent assay (ELISA) kits (Jianglai Bio, Shanghai, China). The protein level of a master gene of autoimmune disease, LACC1 (Jianglai Bio, JL49070), was also measured in the plasma samples by an ELISA kit according to the manufacturer’s instructions.

*Sample preparation* After thawing, the plasma sample from each individual was centrifuged at 4 °C for 10 min at 3000 R/min and then diluted 4 times with an appropriate dilution buffer and used for the analysis of ACA, ANA,ENA, dsDNA, ASO, RF, and LACC1. The ACA, ANA, ENA, dsDNA, ASO, RF, and LACC1 concentrations were determined by commercial ELISA kits (Jianglai Bio, Shanghai, China) according to the manufacturer’s instructions.

*Elisa* Plasma (1:4 diluted with sample buffer, 50 µL per well) and antibodies (marked by HRP, 100μL per well) were add and then incubated at 37 °C for 1 h. Sample wells were washed five times with a provided wash buffer (20 × dilution with ddH2O, 350μL per well), then the provided substrate solution was added (50 µL per well) and incubated at ambient temperature of 37 °C for 15 min. After a second wash step, the provided stop solution was then added (50 μL per well) and absorbance of sample wells measured immediately at 450 nm in 15 min. Data were then analyzed as recommended by the manufacturer.

*Statistical analysis* Comparisons of the level of each autoantibody in different groups were conducted using the nonparametric Mann‒Whitney test. Comparisons of the positive rate of each autoantibody between control and patient groups were conducted using correct Chi-Squared Test of four-fold table, comparisons for five subtypes were conducted using Chi-Squared Test of contingency table. Correlation between the level of autoantibodies and the level of LACC1 protein was measured by Pearson correlation, whereas the alteration of the autoantibodies along age was measured by linear regression. All analyses were carried out with GraphPad Prism (version 8.3.0). *P* values < 0.05 were considered statistically significant.

### Ethics approval and consent participate

The study is approved by the Institutional Review Board of Kunming Institute of Zoology, Chinese Academy of Sciences, with approval number of SMKX-SQ-20200414-075. All subjects participated had providing written informed consent according to the principles of the Declaration of Helsinki. Subjects under 16 years old were not involved in this study. We confirm that all methods were performed in accordance with the relevant guidelines and regulations.

## Data Availability

All data generated or analyzed during this study are included in this published article.
